# The Effect of Feedback on Attention Allocation in Category Learning: An Eye Tracking Study

**DOI:** 10.3389/fpsyg.2020.559334

**Published:** 2020-11-10

**Authors:** Yael Arbel, Emily Feeley, Xinyi He

**Affiliations:** Department of Communication Sciences and Disorders, Massachusetts General Hospital (MGH) Institute of Health Professions, Boston, MA, United States

**Keywords:** category learning, attention allocation, feedback processing, eye-tracking, learning strategies

## Abstract

It has been suggested that category learning involves changes in attention allocation based on the relevance of input to the classification. Using eye-gaze measures, Rehder and Hoffman studied changes in attention allocation during category learning in a 5–4 category structure paradigm with four features of varying diagnosticity levels. In this paradigm, participants are tasked with classifying creatures into two groups through trial and error guided by feedback. While learners’ eye-gaze patterns have been studied as a function of feature diagnosticity levels throughout the learning process, they have not been evaluated in relation to performance and feedback. The present study borrowed and modified Rehder and Hoffman’s category paradigm and evaluated eye-gaze behavior as a function of the diagnosticity level of features, and the valence (positive vs. negative) of the preceding feedback during learning. Our results support Rehder and Hoffman’s observations that gaze on the low diagnosticity feature decreased from the beginning to the end of the task. When change in eye gaze behavior was evaluated in relation to feedback, change in *fixation probability* was found to be greater following negative feedback. The results indicate that in a category task that includes performance feedback, learning strategies as indicated by changes in selective attention to features are affected to some degree by the valence of the feedback on a preceding trial.

## Introduction

Learning involves the ability to selectively attend to stimuli in the environment that contribute to learning (e.g., Restle, 1962; Trabasso et al., 1968). The study of visual selective attention allocation has been enhanced by the ability to capture fine changes in eye-gaze using eye-tracking systems. This methodology has been used to measure attention allocation in the context of visual perception (Loftus and Mackworth, 1978; Henderson, 1999), written language processing (e.g., Just and Carpenter, 1984; Tanenhaus et al., 1995; Rayner, 1998; Mak et al., 2002; Schroeder et al., 2015), social interactions (e.g., Rubo and Gamer, 2018; Jording et al., 2019), and category learning (Rehder and Hoffman, 2005a,b; Zaki and Salmi, 2019), among other contexts. Rehder and Hoffman (2005a) studied attention allocation in category learning using eye-gaze measures. Adopting Shepard et al. (1961) view that strategic allocation of attention is a necessary component of category learning, they examined change in attention allocation to features that were either relevant or irrelevant to the classification. They reported that the number of fixations across features changed from the beginning to the end of the task as a function of feature relevance. The results of their study provided support to the ALCOVE model of category learning which posits that the strength of the associations between exemplars and categories as well as the weighted attentional strength are adjusted through error driven learning that leads to greater attention to dimensions that are more relevant to the category. Rehder and Hoffman (2005b) further examined attention allocation as measured by eye-gaze using a 5–4 category structure as described by Medin and Schaffer (1978). In this task, stimuli belonging to one of two categories varied across four features, with two highly diagnostic features, a moderately diagnostic feature, and a minimally diagnostic one. Eye-tracking data suggested that at the beginning of the task eye-gaze was similar across all features, while toward the end of the task participants looked less at the least diagnostic feature. While no optimal allocation of attention was found, the results demonstrated that participants transitioned to a more efficient attentional strategy over the course of learning.

Although the study of attention allocation in category learning involves the provision of external feedback, the relationship between feedback processing and attention allocation has yet to be evaluated. Learning is often achieved by trial and error guided by external feedback. Such learning involves hypothesis building, testing, and revising based on feedback provided to specific choices. The achievement of optimal learning outcomes in such environment relies on effective processing of feedback. It has been established that humans adjust behaviors based on feedback communicated to them during learning (e.g., Barron and Erev, 2003; Grosskopf et al., 2006; Cohen and Ranganath, 2007; Hattie and Timperley, 2007; Yechiam and Ert, 2007; Erev and Roth, 2014). For example, stay and switch behaviors have been observed in paradigms that include feedback informing learners of gains and losses (e.g., Cohen and Ranganath, 2007). Attention allocation, which has been shown to shift during learning and to be linked to learning outcomes (e.g., Rehder and Hoffman, 2005a; Matsuka and Corter, 2008), is likely to change following feedback to optimize learning. It has also been established that feedback supports category learning (e.g., Freedberg et al., 2017). More specifically, in a study by Freedberg et al. (2017), category learning was enhanced by the provision of negative feedback only or by the provision of both positive and negative feedback, but not by positive feedback alone. Feedback-based learning in the context of category learning is often labeled supervised learning to distinguish it from observational learning which is termed unsupervised. Feedback has a defined role in tuning attention in several models of category learning (e.g., ALCOVE, Kruschke, 1992; SUSTAIN, Love et al., 2004; DIVA, Kurtz, 2007). For example, in the ALCOVE model (Kruschke, 1992) the role of the feedback is to adjust the weights of the associations between the exemplars and the category as well and the attention weight. According to this model, adjustments are driven by errors, suggesting that it is the negative feedback that drives changes in attention weight. In the SUSTAIN model (Love et al., 2004) where perceptual information is translated into a set of features organized in clusters, each associated with a category, the role of feedback is to tune the category structure by recruiting new clusters following misclassifications of input. Therefore, in the SUSTAIN model, which is striving for simplicity but can adjust when the classification turns out to be complex, the feedback plays a role in the adjustment of category representation from simple to complex. Consequently, feedback in the SUSTAIN model alters attention to sets of features based on the revised category representation. Although feedback processing (e.g., Freedberg et al., 2017) and strategic attention allocation (e.g., Nosofsky, 1986; Rehder and Hoffman, 2005a,b, Blair et al., 2009) have been each linked to category learning, the relationship between feedback processing and attention allocation in the context of learning has yet to be examined.

Eye tracking has proven to be a promising method for studying attention allocation in category learning (Rehder and Hoffman, 2005a,b), and has strengthened specific theories of category learning. Given that feedback is an important component in various models of category learning, applying this method to study the effect of feedback on such learning can shed light on the sensitivity of the eye-tracking method to adjustments of attention following positive and negative feedback. If such sensitivity exists, this methodology could enrich the study of the role of feedback in the creation and adjustment of category representations and in improving classification. The present study was designed to examine whether attention allocation as measured by eye-gaze changes as a function of the valence of feedback. The study employed a variation of Rehder and Hoffman’s (2005b) 5–4 classification task, with eye-gaze behavior measured for each of the four features throughout the learning process and in response to feedback provision. In Rehder and Hoffman (2005b), an eye was placed in the “head” feature, defining this feature for the learner as the head, and resulting in greater attention to this perceptually salient feature regardless of its diagnosticity level (Rehder and Hoffman, 2005b). To reduce the effect of perceptual saliency on attention allocation, the eye was removed in the present design. Participants in the current study were not oriented to a feature to be representing a particular body part (i.e., head, tail, wing, and feet). To the best of our knowledge, this is the first study to evaluate the effect of performance feedback on attention allocation as measured through eye-gaze behavior in the context of category learning.

## Materials and Methods

### Participants

Participants were 20 healthy young adults (17 females), ranging from ages 19 to 35 years (*M* = 25.9, *SD* = 3.87). Participants reported no history of developmental or acquired neurological disorders and had normal or corrected to normal hearing and vision. The study was approved by the Partners HealthCare IRB and signed consents were obtained from all participants before data collection was initiated. Data of two participants were removed from the analyses due to difficulties tracking their gaze.

### Experimental Task

**FIGURE 1:**
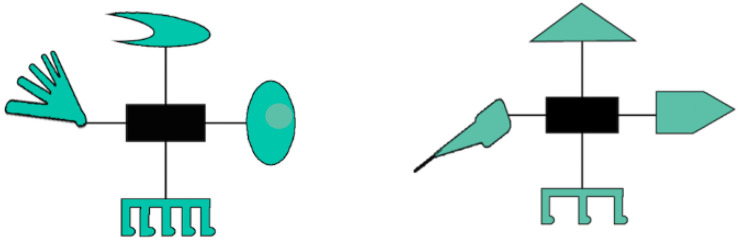
Examples of stimuli belonging to each category. Borrowed from Rehder and Hoffman (2005b).

Participants sat in front of a computer monitor with their head positioned on a chin rest and completed a variation of the Rehder and Hoffman’s (2005b) category learning task while their eye-gaze data were collected. Participants were tasked with learning through trial and error guided by feedback to correctly classify novel creatures presented visually on the screen into one of two categories. The categorization task included a training phase and a test phase. During the training phase, participants were presented with a visual stimulus (see Figure 1) and were asked to classify it as belonging to category A or category B by pressing one of two buttons on a response box. Following the selection, participants received visual feedback consisting of three green checks for correct responses or three red Xs for incorrect responses. The training phase was completed once participants reached an accuracy of 85% on two consecutive blocks, or after 30 blocks of nine trials for a total of 270 trials. The training phase was immediately followed by a test phase, which consisted of the nine stimuli presented during the training phase (trained items), and seven novel stimuli created based on the same categorization structure (transfer items). During testing, participants were asked to categorize stimuli into category A or category B without receiving feedback on their performance. During this phase, stimuli remained on screen until a response was made.

The task utilized Medin and Schaffer’s 5–4 category structure (see Table 1). Stimuli were borrowed from Rehder and Hoffman (2005b) and consisted of 16 creatures, each featuring a head, wings, feet, and tail surrounding a central rectangular body (see Figure 1). Each feature appeared in one of two forms, with each variation assigned a code of 0 or 1. For example, the head was either pointed or oval, and the feet had either three or five toes. Each creature was a combination of the four features in one of their two variations, coded as a set of 0s and 1s (e.g., 1-1-0-0). Each feature was approximately 4° in width and height, and the entire creature was 12° of visual angle in width and height. Similarly to Rehder and Hoffman (2005b), the wings and the feet were the highly diagnostic features (associated with a category in 0.77 of training trials), the head was the moderately diagnostic feature (associated with a category in 0.66 of training trials), and the tail was the minimally diagnostic feature (associated with a category in 0.55 of training trials). The eye was removed from the head feature in the current experiment.

**TABLE 1:** Category membership (category A or B) based on features. The likelihood of a feature to be uniquely associated with a category determined its diagnosticity level (low, moderate, high).

		Dimension diagnosticity		
		
Stimulus	High 1	High 2	Moderate	Low
**Category A items**				
A1	1	1	0	1
A2	1	1	0	0
A3	1	1	1	0
A4	1	0	1	1
A5	0	1	1	1
**Category B items**				
B1	1	0	0	1
B2	0	1	0	1
B3	0	0	1	0
B4	0	0	0	0
**Transfer items**				
T1	1	0	1	0
T2	1	0	0	0
T3	1	1	1	1
T4	0	1	0	0
T5	0	0	1	1
T6	0	1	1	0
T7	0	0	0	1

**FIGURE 2:**
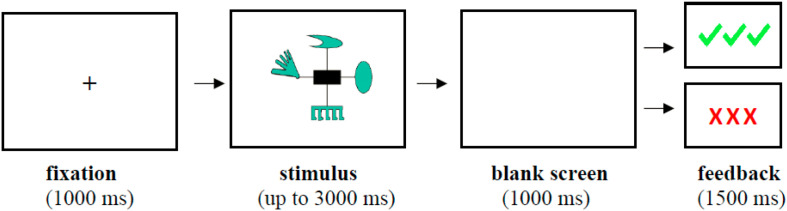
An illustration of a single trial during the training phase of the task.

Each trial started with a fixation point in the center of the screen for 1,000 ms, followed by the stimulus, which appeared for up to 3,000 ms or until the participant responded with a button press to indicate group membership (A or B). Each response was followed by a blank screen for 1,000 ms and with a visual performance feedback for 1,500 ms (see illustration of a trial sequence in Figure 2). If a response was not made within the allotted 3,000 ms, participants were presented with a visual message prompting them to respond faster on the next trials.

### Data Collection and Analysis

#### Behavioral Data

The training phase was divided into three parts based on the total number of trials completed by each participant. Accuracy was evaluated during the training phase for each third (*bin*) of trials, and during the test phase.

#### Eye-Tracking Data

Eye fixations were captured using SR Eyelink 1000 Plus Eye Tracker by SR Research. Monocular eye gaze data were collected after an initial calibration using nine-point calibration and validation. A drift correction, that required participants to fixate a dot at the center of the screen, was performed at the beginning of each block of trials to allow calibration adjustments of the eye-tracker. Four areas of interest were polygons drawn to encapsulate each of the four features. The interest period for each trial was defined as the time from the onset of the stimulus to the participant’s response. A fixation was preceded by a saccade and was determined using a temporal threshold of 100 ms. Two measures of gaze behavior were obtained, *proportion fixation time* and *fixation probability*. To evaluate duration of gaze on each of the features relative to gaze on other features on a given trial, *Proportion fixation time* was calculated by dividing the fixation time on a feature by the total fixation time on the four features in each trial. To measure whether learners looked at a feature within a given trial, *Fixation probability* was calculated by assigning a binary value (0 or 1) to each feature to indicate whether a feature was fixated on at least once during a trial. These measures were used to assess gaze behavior as a function of feature and bin and to evaluate change in gaze behavior over time and in relation to feedback.

Examination of participants’ gaze behavior revealed that 75% of participants focused significantly more on one of the two highly diagnostic features (*High1* and *High2*) than on the other, and that the highly attended-to feature varied across participants. To capture this unique pattern, gaze behavior of the highly diagnostic features was categorized per participant based on the *proportion fixation time* of each of the two features. For each participant, the highly diagnostic feature with the higher proportion fixation time across blocks was labeled *High_High*, and the other highly diagnostic feature was labeled *High_Low*. When averaged across participants, this method of comparison was found to give a more accurate presentation of participants’ gaze behavior across features. A *Bin* by *Feature* ANOVA was conducted to evaluate attention allocation to features throughout the training process as a comparison to Rehder and Hoffman’s (2005b) study.

To examine the extent to which feedback affected eye gaze behavior, change in gaze behavior was evaluated as a function of feedback valance on a preceding trial. To measure change in attention allocation, *change in fixation probability* and *change in proportion fixation time* were obtained for each feature and trial. It was calculated as the absolute value of the difference in *fixation probability/proportion fixation time* between a given trial and the trial preceding it for each feature. C*hange* values were obtained separately for trials following negative and positive feedback and created for each of the three *Bins* to allow the evaluation of change in gaze behavior as a function of feedback and time. A *proportion change* score following each feedback type in each *bin* was calculated as the sum of the change values across features divided by the total number of fixations. Change in attentional allocation was grouped based on *valence* and *bin*, creating six categories: *Bin 1 Positive*, *Bin 1 Negative*, *Bin 2 Positive*, *Bin 2 Negative*, *Bin 3 Positive*, and *Bin 3 Negative*. Repeated measures ANOVAs were completed for *change in fixation probability* and *change in proportion fixation time* with *Bin* and *Valance* as within subject factors.

## Results

### Behavioral Data

Of the 18 participants, whose data were included in the analysis, 7 (35%) reached the learning criterion in fewer than 30 blocks (19–29 blocks). The average accuracy during training was 0.53 (*SD* = 0.08) in *Bin 1*, 0.61 (*SD* = 0.11) in *Bin 2*, and 0.66 (*SD* = 0.12) in *Bin 3*. Repeated measures ANOVA with *Bin* as a within subject variable resulted in a significant effect of *Bin*, *F*(2, 16) = 16.48, *p* < 0.001, η^2^*_*p*_* = 0.49, indicating that accuracy increased throughout the training phase. Pairwise comparison indicated accuracy differences between all bins (*ps* < 0.01). Accuracy on the test phase was 0.71 (*SD* = 0.13). Within the test phase, accuracy on trained items was 0.78 (*SD* = 0.14), while accuracy on transfer items (untrained) was 0.63 (*SD* = 0.17). Repeated measures ANOVA resulted in a significant effect of *Training*, *F*(1, 17) = 13.68, *p* = 0.002, η^2^*_*p*_* = 0.45, indicating that accuracy was greater for trained items.

### Eye-Tracking Data

#### Eye-Gaze as a Function of Features and Bins

##### Proportion fixation time

**FIGURE 3:**
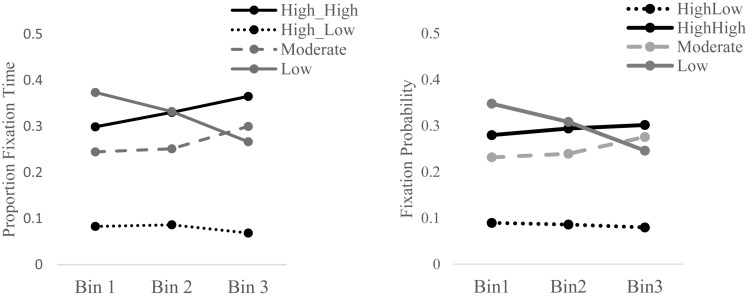
Proportion fixation time (*left*) and fixation probability (*right*) for each of the four features (High_High, High_Low, Moderate, Low) across time (i.e., in each of the three Bins).

A repeated measures ANOVA of *proportion fixation time* revealed a main effect of *Feature*, *F*(3, 15) = 6.85, *p* = 0.003, η^2^*_*p*_* = 0.28, indicating that fixation time was not equal across features. *Post hoc* pairwise comparisons indicated that the less attended to of the two highly diagnostic features (*High_Low*) was associated with significantly less fixation time than the other three features (*ps* < 0.0001). It is important to note that the *High_Low* feature was, by definition, associated with lower fixation time than the *High_High* feature. No other differences in proportion fixation time were found between features of different diagnosticity level. While no effect of *Bin F*(2, 16) = 3.17, *p* = 0.09 was found, an interaction between *Bin* and *Feature* was detected, *F*(3, 15) = 2.88, *p* = 0.04, η^2^*_*p*_* = 0.16. *Post hoc* comparisons indicated that this interaction was driven by differences in proportion fixation time between the first and the last *bins* only in relation to the *low* diagnostic feature (*Low* diagnostic feature *Bin 1* vs. *Bin 3*, *t*(17) = 3.04, *p* = 0.007). These results point to a reduction in *proportion fixation time* on the *low* diagnostic feature from the first to the third bin of the training phase (see Figure 3, left).

##### Fixation probability

A repeated measures ANOVA of *fixation probability* revealed a main effect of *Feature*, *F*(3, 15) = 7.46, *p* = 0.002, η^2^*_*p*_* = 0.3, indicating that fixation probability was not equal across features. Pairwise comparison indicated that the less attended to of the two highly diagnostic features (*High_Low*) was associated with significantly less fixation time than the other three features (*ps* < 0.001). No effect of *Bin*, *F*(2, 16) = 3.34, *p* = 0.054, η^2^*_*p*_* = 0.16, or an interaction of *Bin* and *Feature*, *F*(6, 12) = 2.03, *p* = 0.123, η^2^*_*p*_* = 0.1, were found (see Figure 3, right).

#### Change in Eye-Gaze Behavior as a Function of Feedback Valence and Bins

Change in eye-gaze behavior was calculated based on two measures, the proportion fixation time, and fixation probability as described above. The proportion change in fixation time and fixation probability as a function of bin and feedback is presented in Table 2.

**TABLE 2:** Proportion change in fixation time and fixation probability following positive and negative feedback in each of the three training bins.

		Following positive feedback Mean (SD)	Following negative feedback Mean (SD)
Change in fixation time	Bin 1	0.72 (0.20)	0.80 (0.20)
	Bin 2	0.79 (0.26)	0.81 (0.31)
	Bin 3	0.90 (0.33)	0.89 (0.33)
Change in fixation probability	Bin 1	0.52 (0.22)	0.56 (0.18)
	Bin 2	0.54 (0.27)	0.59 (0.36)
	Bin 3	0.61 (0.36)	0.65 (0.37)

##### Change in proportion fixation time

A repeated measures ANOVA of *change in proportion fixation time* as a function of *Bin and Valence* resulted in a main effects of *Bin, F*(2, 16) = 3.7, *p* = 0.03, η^2^*_*p*_* = 0.18, suggesting a gradual increase in change from Bin 1 to Bin 3. No effects of *Valence, F*(1, 17) = 2.12, *p* = 0.16, η^2^*_*p*_* = 0.11, or an interaction between *Bin* and *Valance F*(2, 16) = 1.67, *p* = 0.2, η^2^*_*p*_* = 0.09. These results suggest that change in fixation duration (i.e., change in how long learners gazed at features) increased during the learning process (more change in Bin 3) but was not affected by the valence of the feedback.

##### Change in fixation probability

**FIGURE 4:**
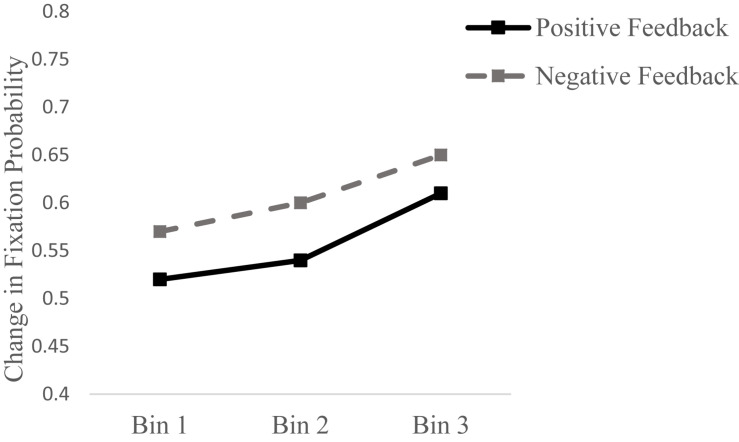
Proportion change in fixation probability following positive (black solid line) and negative feedback (gray dashed line) in each of the three bins.

A repeated measures ANOVA of *change in fixation probability* as a function of *Valence* and *Bin* resulted in no effect of *Bin*, *F*(2, 16) = 1.75, *p* = 0.19, η^2^*_*p*_* = 0.09, but revealed a main effect of *Valence, F*(1, 17) = 6.53, *p* = 0.02, η^2^*_*p*_* = 0.27, indicating that change in fixation probability was greater after negative feedback (see Figure 4). No interaction between *Bin* and *Valance* was found, *F*(2, 16) = 0.03, *p* = 0.96, η^2^*_*p*_* = 0.002. These results indicate that change in fixation probability (i.e., change in whether a feature was fixated on) was greater after negative feedback throughout the learning process.

## Discussion

The study aimed at evaluating the effect of performance feedback on attention allocation during category learning. Learning was evaluated within a context of a category learning task that was borrowed from Rehder and Hoffman (2005b) and modified. An initial evaluation of the eye-gaze behavior in the present study was done in comparison with the findings of Rehder and Hoffman (2005b) to determine the extent to which the modifications employed by the present study affected patterns of attention allocation in this learning paradigm. We then examined the extent to which change in eye-gaze behavior was associated with the valence of the feedback on a preceding trial.

### Comparison to Rehder and Hoffman

The results reported by Rehder and Hoffman (2005b) indicated that attention allocation as measured by eye-gaze changed over the course of the learning process as a function of the diagnosticity level of the features, with the least diagnostic feature being less attended to than the moderately diagnostic feature as learning progressed. In line with Rehder and Hoffman’s (2005b) report, our results indicated a reduced fixation duration to the low diagnostic feature from the beginning to the end of the learning paradigm. The pattern of reduced attention to the low diagnostic feature throughout the learning process did not reach significance when fixation probability served as a measure of eye-gaze behavior. This discrepancy between the two measures (*proportion fixation time* and *fixation probability*) suggests that although participants learned to look for shorter durations at the low diagnostic feature, they still gazed at this feature. It is possible that the lack of change in fixation probability from the first bin to the last stems from the engagement of the participants in learning even toward the end of the task. This suggestion is supported by the relatively low accuracy rate of the participants (mean of 0.68) on their last training bin. One possible contributor to the lower learning outcomes in the current study is shorter stimulus duration. In Rehder and Hoffman’s study (2005b) each stimulus was displayed for four additional seconds following the participants’ response and the proceeding performance feedback, while in the present study, stimuli were removed immediately following the response to separate the processing of the stimuli from the processing of the feedback. The removal of the eye from the stimuli in the present study could have also contributed to learning outcomes by eliminating the possible facilitating effect of feature saliency on learning, and by altering the participants’ orientation to the stimuli.

The present study yielded a surprising pattern of attention allocation to the two highly diagnostic features such that participants tended to focus the least on one of the highly diagnostic features throughout the learning paradigm. This pattern has not been observed or reported in Rehder and Hoffman (2005b). It is possible that the learners in the current study realized early in the learning process that the two highly diagnostic features were more likely to appear together than other features (i.e., the two highly diagnostic features appeared together on 60% of the items belonging to category A during training). While this strategy may have contributed to overall accuracy during training, it was a likely contributor to relatively low accuracy rate on the transfer items on the test in which the two highly diagnostic features appeared together only in 43% of the trials. Interestingly, Rehder and Hoffman (2005b) reported that while their results indicated suboptimal attention allocation when using the 5–4 structure, unpublished work in their lab, using three rather than four features, produced results that matched the original pattern of optimized attention observed by Shepard et al. (1961). It is unclear, though, whether all three features had different diagnosticity levels (i.e., low, moderate, high as oppose to two features with similarly high diagnosticity levels). The suboptimal attention allocation observed in the present study and in Rehder and Hoffman (2005b) can be attributed to the nature of the task where classification is done based on spatially separated dimensions that are assumed to gain unique attentional advantage over time based on their contribution to the classification. This assumption may be challenged by the notion that features that are grouped together, gradually form a perception of a whole (Goldfarb and Treisman, 2010, 2013; Goldfarb and Sabah, 2016). Goldfarb and Sabah (2016) present the *association effect* as the cost in reaction time when binding a feature and an object that are inconsistently associated, and propose a process within the framework of the object file theory that allows the binding of such features with an object. It is important to note that unlike the 5–4 paradigm where features differ in their relevance to the categorization, the features in Goldfarb and Treisman (2013) were equally relevant to the representation of the object. We suggest that the relevance of the feature to the representation of an object or category affects the extent to which it is included in the binding of the features.

### The Effect of Feedback Valance on Change in Attention Allocation

Change in attention allocation was calculated based on two eye-gaze measures, *proportion fixation time* and *fixation probability*. The degree of change in how long participants looked at features (*change in proportion fixation time*) was not found to be related to the valence of the feedback. However, the change in whether a feature was being looked at (*change in fixation probability*) was found associated with feedback valence, such that greater change occurred following negative feedback than following positive feedback. These results indicate that while “looking time” was not affected by feedback, the choice of which features to look at changed as a function of the valence of the feedback, with negative feedback leading to greater change in feature choice. The lack of change in proportion fixation time following negative feedback is not surprising as participants are expected to remain engaged with the stimuli before learning is established. It is possible that “looking time” did not change following positive feedback because our participants have not achieved a conscious mastery of the categorization and continued to use positive feedback to inform their decisions instead of to confirm their knowledge. The increased change in proportion fixation time from the beginning to the end of the task, regardless of feedback valence may indicate that participants used both positive and negative feedback to inform their learning, and that toward the end of the task they gained more control over the task as indicated by growing change in their gaze behavior. Our finding that *change in fixation probability* (a change in whether a feature was being looked at) was greater following negative feedback than positive feedback suggests that participants may have modified their learning strategy more following negative feedback. This behavior is in line with findings of strategy adjustment following negative feedback (e.g., Cohen and Ranganath, 2007).

## Limitations and Future Directions

The relatively low accuracy rate obtained in the present study suggests that data captured in this experiment does not account for the complete learning process but rather reflects processes associated with earlier-mid stages of learning. We suggest that with additional training blocks, participants would have achieved higher learning outcomes and the patterns of learning as measured by eye-gaze would have shown more robust patterns. Nonetheless, the observed patterns of the present study are in line with those reported by Rehder and Hoffman (2005b). Our observation that participants tended to ignore one of the two highly diagnostic features which were highly correlated during training was surprising. This pattern may have contributed to the relatively low accuracy rates. Future examinations of the effect of diagnosticity level on attention allocation in category learning should assign a distinct probabilistic value to each feature so that there is one feature per level. It is worth noting that the two highly diagnostic features were positioned in the vertical plane (below and above the body), whereas the other two features were on the horizontal plane. The position of the features may have affected eye gaze behavior. For example, it is possible that gaze was biased toward a horizontal scanning which is often used in reading text. Future studies should consider varying the positions of features of different diagnosticity levels to control for this possible bias. In the present study, the eye was removed from the head feature to reduce the effect of feature saliency on attention. However, feature saliency cannot be overlooked as a possible factor affecting attention allocation in the present study as learners still perceived the stimuli as “creatures” and may have attended more to the feature they have denoted as the head. Gaining insight into the learners’ perception could be achieved through a follow up questionnaire. Such questionnaire could also shed light on the learners’ conscious response to positive and negative feedback during training.

## Data Availability Statement

The raw data supporting the conclusions of this article will be made available by the authors in accordance with the process set by the first author’s institute.

## Ethics Statement

The studies involving human participants were reviewed and approved by the Partners IRB. The participants provided their written informed consent to participate in this study.

## Author Contributions

YA provided training and oversight related to data collection and analysis, contributed to data analysis, and led the writing of the manuscript. EF collected the data and contributed to data analysis. XH performed additional analysis of eye-tracking data and statistical analysis. All authors contributed to the article and approved the submitted version.

## Conflict of Interest

The authors declare that the research was conducted in the absence of any commercial or financial relationships that could be construed as a potential conflict of interest.
